# Survival Following Rectal Impalement through the Pelvic, Abdominal, and Thoracic Cavities: A Case Report

**DOI:** 10.1155/2009/361829

**Published:** 2009-07-01

**Authors:** Michael Moncure, Jared A. Konie, Adam B. Kretzer, Peter J. DiPasco, Carla C. Braxton

**Affiliations:** University of Kansas Hospital, 3901 Rainbow Boulevard, Kansas City, KS 66160, USA

## Abstract

Impalement injuries are a unique form of penetrating trauma and are typically associated with a fall onto the object (Steele, 2006). We present the case of a 45-year-old man who reportedly slipped in his bathtub and fell onto a broomstick. Radiographic examination revealed a slender mass extending from his rectum to the right side of his neck. A review of English literature suggests that this is the second reported case in the last 100 years describing the successful management of an impalement injury traversing the pelvic, abdominal, and thoracic cavities. The management of this case is described.

## 1. Case Report

**Figure 1:**
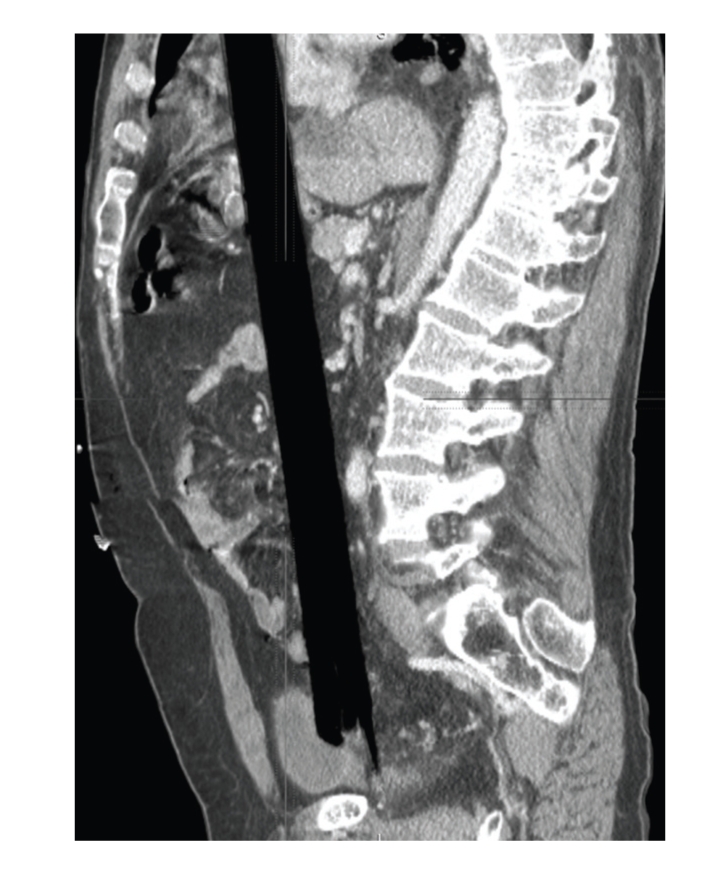
Sagittal CT radiograph showing the splintered end of the impaled object.

**Figure 2:**
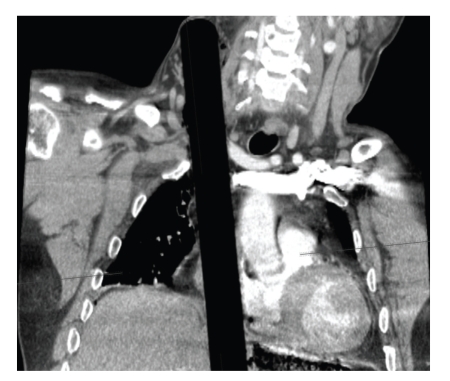
Coronal CT radiograph showing the blunt end of the object extending into the neck.

**Figure 3:**
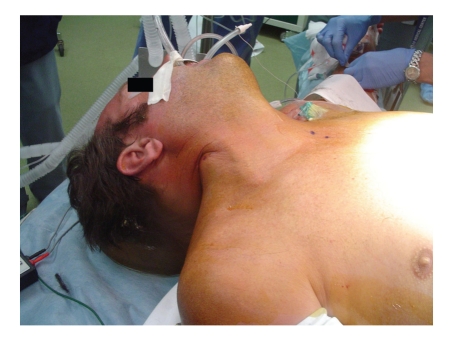
Photograph showing the object tenting the right side of the patient's neck.

**Figure 4:**
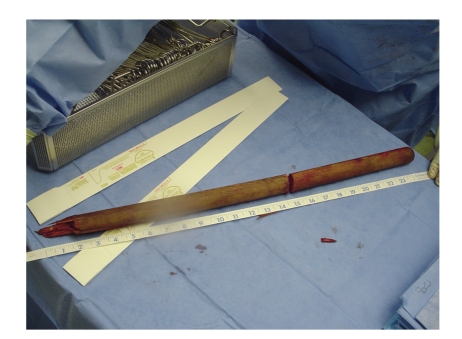
The extracted wooden rod measured 63.5 cm in length.

Upon arrival to our ACS Level I Trauma Center and teaching hospital, the patient was alert and hemodynamically stable with the chief complaint of abdominal pain. After trauma evaluation and assurance of his stability, radiographic images (plain X-rays and CT scans) were obtained (Figures 1  and 2) and the decision was made to bring the patient to the operating room for formal exploration and operative management of his injuries. On exploration via median sternotomy and midline laparotomy it was discovered that the impaling object was a blunt-ended wooden rod 3 centimeters in diameter with its splintered end within the rectum. It pierced the anterior intraperitoneal rectum, traversed the small and large bowel mesentery three times, passed through-and-through the stomach, pierced the diaphragm and pericardium, and laid with its distal end pinned below the right clavicle and extending into the neck (Figure 3). Surprisingly, there were no vascular injuries along its track, presumably due to its rounded end acting as a blunt tunneler. To remove the offending object, it was decided to sever the rod within the mediastinum using a bone saw and remove the abdominal portion antegrade. A second incision was made above the prominence in the right anterior neck triangle and the superior segment was pulled antegrade through this orifice. The wooden rod's overall length was 63.5 centimeters (Figure 4). Primary repairs were performed on the diaphragmatic and gastric injuries, mesenteric tears, and the rectal perforation. Due to the extensive nature of the patient's injuries and high suspicion of ensuing intraperitoneal sepsis, a Hartmann's Procedure was performed, and the pelvis was excluded from the rest of the abdominal cavity by closing the overlying peritoneum. Thoracostomy and mediastinostomy tubes were placed, as well as intra-abdominal drains. Total operative time from primary incision to closure was 3 hours 52 minutes. Surgeons included a trauma surgeon, a cardiothoracic surgeon, and three surgery residents.

Postoperatively in the surgical intensive care unit, the patient's pain was managed with a PCA pain pump and nutrition was provided following NG tube placement. The patient experienced a new onset of atrial fibrillation and was treated with amiodarone and beta blocking agents. Two weeks postoperatively, his stay was further complicated by a hospital-acquired pneumonia that progressed to an episode of severe sepsis. Following antibiotic treatment with a full 24-hour surviving sepsis bundle, multiple line placements, and a bronchoscopy and tracheostomy, his sepsis was resolved. Other complications included right upper extremity weakness due to complete brachial plexopathy, a ventral incisional hernia, and a loculated right effusion resulting in a prolonged vent weaning period. Two months after his admission, the patient was discharged in good functional status to our inpatient rehabilitation facility, and he returned home a week later. This patient has since had his colostomy reversed and is doing well. Colostomy reversal and herniorrhaphy were performed 17 months after his initial injury, and the patient underwent a second hernia repair procedure 1 year later. All together, this patient was brought to the operating room 3 times over a span of approximately 2.5 years.

## 2. Discussion

Ninety-five percent of colonic injuries seen in urban trauma centers are penetrating gunshot and stab wounds; the remaining 5% are the result of blunt trauma or transanal injury [1]. Transanal injuries that are not iatrogenic are most often caused by a myriad of sexually related activities which can be quite severe and have even been lethal [1–5]. Unfortunately, patients who sustain such sexually related anorectal injuries often delay seeking medical attention and deny the circumstances surrounding the injury [6]. In the pediatric population, sexual abuse is the number one cause of isolated rectal injuries and should always be suspected until shown otherwise [6, 7].

Many currently accepted treatments for penetrating anorectal and bowel injuries such as diverting colostomies and presacral drains evolved from several decades of war time experience [8–11]. The success of these treatments in noncombat trauma situations however has been questioned, and many recommend an evidence-based approach based upon precise anatomic injury location rather than performing procedures simply because they have been the standard for so long [12, 13]. For the clinical scenario presented in this report, end colostomy was chosen in consideration of the nature of the injuries and the innumerable foreign bodies within the abdomen in the form of wood splinters.

The vast majority of diaphragmatic ruptures are penetrating and are not treated with a combined abdomino-thoracic surgical approach [14, 15], however this was necessary to assess the extent of injuries sustained and manage any rapid exsanguination that could occur upon release of tamponade of the great vessels following dislodgement of the foreign body. There are currently no clear guidelines for the operative management of extensive impalement injuries due in large part to the great variance in impalement mechanisms with which patients may present. We recommend removal of impaled objects utilizing a multimodality surgical approach in a tertiary care facility under the guidance of surgeons specialized in the particular anatomical regions and systems affected by the objects. In the case of our patient, a trauma surgeon and a cardiothoracic surgeon were able to treat the multiple injuries he sustained.

Impalement injuries have been documented since the early beginnings of recorded history in ancient Egypt [16]. Throughout the medieval period rectal impalement as a means of torture and execution became common practice, particularly among the Ottoman Turks [17, 18]. Perhaps the most famous rectal impalement case is illustrated in Nobel Prize winning author Ivo Andric's novel *The Bridge on the Drina * [19]. In his book, Andric describes an executioner hammering an oak stake through a man's rectum, precisely guiding it “in the right direction,” consciously careful not to harm “any of the more important internal organs,” until it finally exited superiorly “close to the right shoulder muscle,” “level with the right ear,” and “had not seriously damaged the intestines, the heart, or the lungs” [19]. Proper placement of the stake was intended to prolong suffering by lengthening the victim's postimpalement survival time. Although this type of impalement did take place throughout the middle ages, the particular execution Andric describes is entirely fictitious from a historical standpoint [17]. Nonetheless, his description of the impaled object coursing superolaterally to the right through the pelvic, abdominal, and thoracic cavities, as well as his depiction of the injuries the victim sustained, is likely accurate as evidenced by the survival of the patient presented in our report.

The impaled object in our patient passed through the entire length of his torso yet did not shear any major blood vessels or permanently damage any organs. A similar anorectal impalement injury was reported in 1981 where a 26-year-old woman jumped from a second story window of a burning building and landed on a tree [20]. The tree branch entered the anus, traveled superiorly to the right perforating mesentery and lacerating the right lobe of the liver antero-inferiorly, split the costochondral junction between the eighth to tenth ribs on the right side, and finally exited through the areola [20]. As was the case with our patient, she was discharged from the hospital with a temporary colostomy after 8 weeks.

## 3. Conclusion

This patient survived a complicated and potentially lethal penetrating injury. Like other similar bowel perforating injuries, this case was managed with a colostomy and resulted in no intra-abdominal infectious complications. The anatomic location of the impaled object was fortunate, and minimal manipulation of the object was critical in order to preserve any potential tamponade effects and minimize blood loss. His current level of health supports both historical and evidence-based treatment protocols. Through the cooperative efforts of trauma, cardiovascular, and orthopedic surgeons, specialists in cardiology, neurology, and infectious disease, and dozens of support staff members our patient experienced a successful outcome.
